# VHL inactivation without hypoxia is sufficient to achieve genome hypermethylation

**DOI:** 10.1038/s41598-018-28795-y

**Published:** 2018-07-13

**Authors:** Artem V. Artemov, Nadezhda Zhigalova, Svetlana Zhenilo, Alexander M. Mazur, Egor B. Prokhortchouk

**Affiliations:** Institute of Bioengineering, Research Center of Biotechnology RAS, Moscow, Russia

## Abstract

VHL inactivation is a key oncogenic event for renal carcinomas. In normoxia, VHL suppresses HIF1a-mediated transcriptional response, which is characteristic to hypoxia. It has previously been shown that hypoxic conditions inhibit TET-dependent hydroxymethylation of cytosines and cause DNA hypermethylation at gene promoters. In this work, we performed VHL inactivation by CRISPR/Cas9 and studied its effects on gene expression and DNA methylation. We showed that even without hypoxia, VHL inactivation leads to hypermethylation of the genome. Hypermethylated cytosines were evenly distributed throughout the genome with a slight preference for AP-1 (JUN and FOS) binding sites. Hypermethylated cytosines tended to be enriched within the binding sites of transcription factors that showed increased gene expression after VHL inactivation. We also observed promoter hypermethylation associated with decreased gene expression for several regulators of transcription and DNA methylation including SALL3.

## Introduction

Sequencing of cancer genomes was initially aimed to find cancer drivers, or genes, that, once mutated, give a selective advantage to a cancer cell, such as increased proliferation, suppression of apoptosis or the ability to avoid immune response. VHL is a key tumour suppressor gene for kidney cancer. Inactivation of the VHL gene is the most common event in renal carcinomas, accounting for 50–70% of sporadic cases^1–3^. Other drivers frequently mutated in kidney cancer include SETD2, a histone methylase which specifically trimethylates H3K4me3.

Tumor cells were shown to have altered DNA methylation profiles compared to normal cells of the same origins. Promoter DNA methylation can cause transcriptional inactivation of certain tumour suppressors, including the VHL gene in renal cell carcinomas. This event is mutually exclusive with VHL inactivation caused by deleterious mutations^1,2^.

VHL gene is a key regulator of hypoxia. VHL is known to direct poly-ubiquitylation and further degradation of HIF1a, a transcription factor which activates hypoxia-associated genes^4,5^. Therefore, inactivated or mutated VHL causes the accumulation of HIF1a transcription factor and subsequent activation of hypoxia transcription program. Interestingly, hypoxia itself is an important oncogenic factor: presence of hypoxia within a tumor is known to be associated with increased tumour malignancy, metastases rate and reduced overall survival^6^. Previously, we have shown that frameshift mutation at the C-terminus of the VHL gene leads to Hif1a stabilization followed by activation of a number of hypoxia-associated genes. VHL inactivation did not affect the rate of cell division, but increased cell viability after reaching the confluence: while the cells with wild-type VHL started dying after reaching the confluence, no such effect was observed for the cells with inactivated VHL^7^.

DNA methylation is maintained and regulated by an oxidation-reduction reaction. The major DNA demethylation mechanism involves oxidation of methylcytosines to hydroxymethylcytosines with Tet enzymes which essentially are oxidoreductases that depend on Fe^2+^ and alpha-ketoglutarate^8^ and can be affected by an altered oxidation-reduction potential in hypoxia. It has recently been shown that DNA methylation is in fact affected under hypoxia^9^. The levels of hydroxymethylated cytosine, an intermediate during cytosine demethylation, were drastically decreased in hypoxia. The CpGs which would normally be hydroxymethylated and then demethylated, failed to do so under hypoxia due to reduced Tet activity. Interestingly, these CpGs were mainly localized within promoters of important tumour suppressors thus preventing these genes from being transcriptionally activated. Reduced activity of hydroxymethylation process was also shown to cause an expected increase in overall DNA methylation at promoters.

In this work, we inactivated VHL gene in a renal carcinoma cell line Caki-1 with CRISPR/Cas9 editing to find out which consequences this common oncogenic event directly caused for gene expression and DNA methylation.

## Results

### VHL gene editing

**Figure 1:**
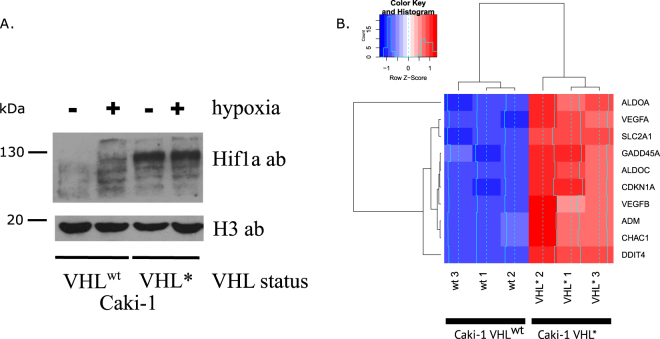
(**A**) Western blot depicting the levels of Hif1a and H3 (control) proteins depending on VHL mutation status and the presence of hypoxia. (**B**) Heatmap showing the changes in expression of the hypoxia-associated genes. Hypoxia-inducible genes were activated in Caki-1 VHL* cells.

We applied a CRISPR/Cas9 assay to introduce a homozygous frameshift deletion into VHL gene. This frameshift led to a premature stop in the C-terminal alpha domain of VHL. We checked how VHL editing affected the stability of Hif1a protein. In fact, Hif1a levels dramatically increased after VHL inactivation (Fig. 1A).

### VHL inactivation caused transcriptional upregulation of hypoxia-associated genes

We first checked if VHL inactivation led to a transcriptional response similar to that caused by hypoxia conditions. We took a conventional set of genes which were associated with hypoxia^10,11^ and explored their expression in VHL wild type Caki-1 (further referred to as Caki-1 VHL^wt^) cells and in VHL* cell lines after VHL editing (further referred to as Caki-1 VHL*). All of the considered genes showed significant upregulation (Fig. 1B), which corresponded to our findings previously obtained by RT-qPCR for a different clone with VHL gene inactivated by editing^7^.

### VHL inactivation caused overall genome hypermethylation in cell lines and tumours

**Figure 2:**
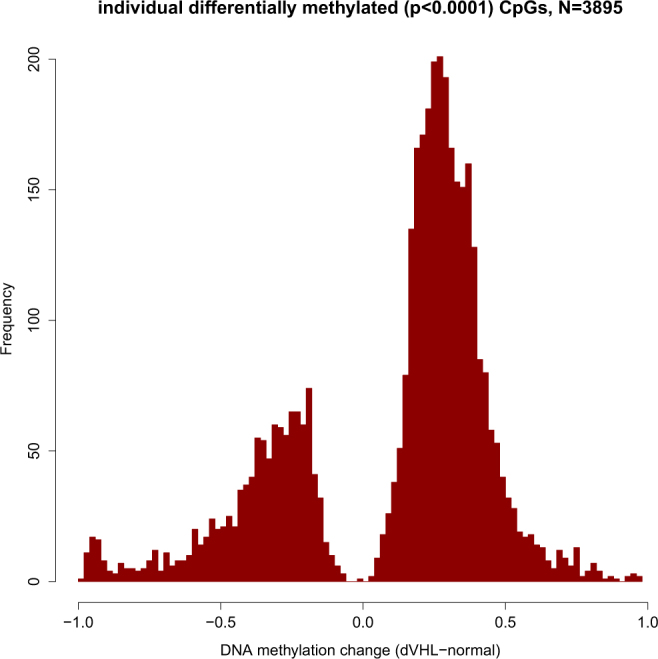
Distribution of changes in DNA methylation rates at individual CpGs following VHL inactivation. Only CpG positions which significantly changed their DNA methylation level were considered. DNA is on average hypermethylated after VHL inactivation.

We compared DNA methylation between VHL-wild type Caki-1 cell lines and the clone of Caki-1 cell line (Caki-1 VHL^wt^) with VHL gene inactivated by a frameshift introduced by CRISPR-Cas9 gene editing (Caki-1 VHL*). We asked how DNA methylation of individual CpG dinucleotides was affected by VHL inactivation. Surprisingly, we observed a dramatic increase in genomic DNA methylation: most of the CpGs which significantly changed DNA methylation appeared to increase it (Fig. 2).

To prove that the observed effect of genome hypermethylation was not specific to the studied cell line, we performed similar analysis in four NCI60 renal cancer cell lines. We compared DNA methylation between renal cancer cell lines with mutated VHL (A498, 786-O) and those with wild-type VHL (ACHN, Caki-1). Similarly to our findings, DNA methylation was significantly higher in cell lines with mutated VHL (*P* < 2 * 10^−16^ for all CpGs, Figure S1A).

As VHL mutation is the most common driver of kidney cancer, we asked if elevated DNA methylation was a feature of tumours with mutated VHL. We excluded tumours with mutated SETD2 gene as this mutation was known to be associated with altered epigenetic phenotype. Indeed, TCGA kidney tumours with mutated VHL show increase in DNA methylation as compared to kidney tumors with wild-type VHL (*P* < 2 * 10^−16^ for all CpGs, Figure S1B). Taken together, the results in four NCI60 cell lines and in TCGA tumours support the hypothesis that VHL inactivation is associated with global genome hypermethylation.

### Hypermethylated DMRs were associated with chromatin modifiers

**Figure 3:**
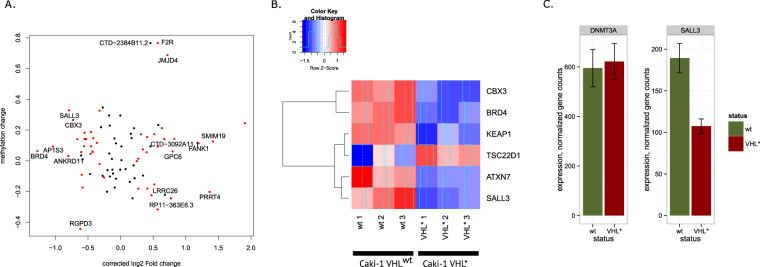
DMRs associated with gene TSSs. (**A**) Relation between gene expression fold-change and the change in DNA methylation within a DMR located near gene TSS. Gene names are shown for the genes with high changes in expression or promoter DNA methylation. (**B**) Gene expression of transcription-related genes which were associated with a significantly hypermethylated DMR. (**C**) Gene expression of SALL3, known to interact with DNMT3A, is significantly decreased in Caki-1 VHL* cells. Expression of DNMT3A itself was not affected.

We discovered differentially methylated regions (DMRs) associated with VHL inactivation. Among 125 discovered DMRs, 86 significantly increased and 39 significantly decreased DNA methylation in VHL* Caki-1 cells. The vast majority of the DMRs (81 out of 125 DMRs) was associated with transcription start sites (TSS) of known genes. Figure 3A shows what kind of gene expression changes happen in the genes associated with a DMRs. Overall, a trend associating increased promoter methylation with decreased gene expression can be observed, though the effect is not significant due to several outliers. We studied which gene categories (GO) were enriched by the genes associated with the DMRs. Interestingly, the DMRs hypermethylated in VHL* Caki-1 cells were associated with genes encoding transcription factors (GO categories Transcription regulation, Transcription, Nucleotide binding). Moreover, we showed that expression of the six genes each falling in those categories was decreased after VHL inactivation (Fig. 3B). Among the genes with the strongest decrease in transcription and increase in promoter methylation in VHL* Caki-1 cells, we discovered SALL3, which was known to interact with DNMT3A and reduce DNMT3A-mediated CpG island methylation^12^. Therefore, the decrease of SALL3 expression (with no change in DNMT3A expression, Fig. 3C) could potentially contribute to genome hypermethylation.

### AP-1 binding sites were enriched with hypermethylated CpGs

**Figure 4:**
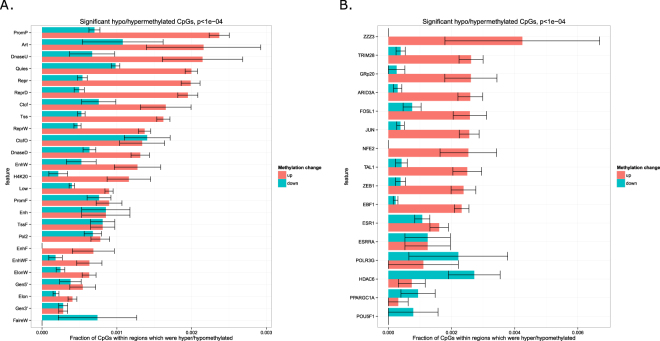
Distribution of hypo- and hypermethylated CpGs within certain epigenomic features. Fraction of significantly hypo- (blue bars) and hypermethylated (red bars) CpGs among all CpGs within a given set of genomic regions. (**A**) Regions of ChromHMM genome segmentation. (**B**). Transcription factor binding sites (only the sites with the highest hyper- and hypomethylation rates are plotted). CpGs that were significantly hypermethylated after VHL inactivation, were enriched in AP-1 (JUN/FOS) and TRIM28 binding sites. Hypomethylated CpGs were enriched in HDAC6 binding sites.

To attribute the observed DNA methylation changes to certain epigenetic mechanisms, we explored DNA methylation changes within different genomic and epigenomic markups. For each set of genomic intervals, we calculated a fraction of significantly hypo- or hypermethylated CpGs among all CpGs located within a given set of genomic intervals for which DNA methylation level had been profiled in the experiment. We started with genome segmentation performed by ChromHMM which subdivides genome into non-overlapping regions representing chromatin state (e.g., promoter or enhancer) according to the data on multiple epigenetic features. The highest fraction of hypermethylated CpGs was observed in inactive promoters (PromP) and weak or unconfirmed enhancers (DnaseU), while active enhancers show rather unchanged DNA methylation pattern with equal rate of hypo- and hypermethylation (Fig. 4A).

To understand if the overall genome hypermethylation could be caused by transcription factors, particularly by those involved in hypoxia response programme, we considered a comprehensive set of transcription factor binding sites annotated by ENCODE project (Fig. 4B, full version in Figure S2). We also included occurrences of HIF1a binding motif as HIF1a level was increased in VHL* cells. DNA methylation changes within HIF1a domains were not different from that observed genome-wide. Interestingly, the highest fraction of hypermethylated CpGs was observed in TRIM28 and AP-1 (JUN and FOS) binding sites, while only in HDAC6 binding sites we observed hypomethylation rather than hypermethylation.

**Figure 5:**
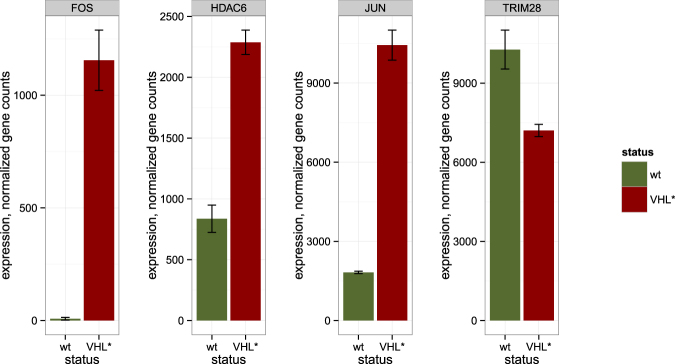
Gene expression in wild type Caki-1 and Caki-1 VHL* cells for certain genes either involved in regulation of DNA methylation or having their binding sites enriched with hypomethylated or hypermethylated CpGs.

To investigate how the changes of DNA methylation in the binding sites corresponded to gene expression changes of the respective transcriptional factors, we studied gene expression of FOS, JUN, TRIM28 and HDAC6 (Fig. 5). All of the considered genes had significant expression changes according to DESeq2 test. Even though the genes showed different direction of expression changes, an apparent pattern could be observed: overexpression of activators (such as JUN and FOS genes forming together AP-1 transcription factor) and decreased expression of repressors (such as TRIM28) caused an increase in DNA methylation at their respective sites, while overexpression of a histone deacetylase HDAC6, involved in epigenetic repression, caused a decrease in DNA methylation at its sites. Indeed, DNA binding proteins with more than twofold increase in expression (in Caki-1 VHL* cells compared to Caki-1 VHL^wt^) show higher than average level of hypermethylation in their sites (Figure S3AB). Therefore, the regions which could become more active due to an overexpression of a transcriptional activator or a decreased expression of transcriptional suppressor were preferred targets of DNA hypermethylation. This pattern can be explained by a hypothesis that DNA methylation in active chromatin is more prone to the processes causing DNA hypermethylation in VHL* Caki-1 cells.

## Discussion

It has recently been proposed that hypoxic conditions themselves can affect the functioning of the TET enzyme that is essentially an oxidoreductase^9^. Low oxygen concentrations caused the decrease of hmC levels and subsequently led to overall hypermethylation at certain genomic loci. This effect was claimed to be independent of hypoxia signaling pathways, but rather was biochemically associated with decreased redox potential in hypoxia. Therefore, it was important to investigate if similar effect could be observed in conditions which are similar to hypoxia in terms of gene activation and cell signalling, but different due to the absence of actual hypoxia. VHL inactivation can be a model of such conditions and also represents an important oncogenic event. Interestingly, we observed overall genome hypermethylation even without hypoxia. Importantly, the changes in DNA methylation in TCGA tumours were similar to those observed in our model system: tumours having mutated VHL had increased DNA methylation compared to those with wild-type VHL. We observed similar differences for kidney cancer cell lines with inactivated VHL as compared to cell lines with wild-type VHL. Further studies are required to reveal if VHL mutation and subsequent activation of glycolysis-related pathways could affect the levels of α-ketoglutarate, α-hydroxyglutarate and succinate and thus impair TET-based demethylation that would subsequently result in genome-wide hypermethylation^13^.

The regions with significantly altered DNA methylation in VHL* cells were located at transcription start sites of the genes which were transcription factors and chromatin modifiers. The expression of these genes, apart of some outliers, is anticorrelated with DNA methylation. Moreover, SALL3, the gene with the strongest concordant increase in promoter methylation and decrease in expression, was known to reduce DNA methylation^12^. Therefore, decreased expression of SALL3 can be one of the mechanisms of the observed genome hypermethylation. Interestingly, hypermethylation of SALL3 promoter has been shown to be associated with poor survival in head and neck cancer^14^. This behaviour of SALL3 can arguably be an example of a positive feedback loop in which expression of a certain epigenetic modifiers is, on one hand, affected by DNA methylation and, on the other hand, influence genomic DNA methylation.

In an attempt to attribute the changes of DNA methylation to certain genomic and epigenetic features, we discovered that, among other transcription factor binding sites, the highest rate of hypermethylation was observed in AP-1 (Jun/Fos) binding sites. Surprisingly, Jun and Fos genes showed increased gene expression after VHL inactivation. It could be expected that this would lead to activation of their targets and subsequent decrease in methylation around their sites. However, an opposite effect was observed. To explain this contradiction, we formulated a hypothesis that DNA hypermethylation occurred genome-wide, but the regions of more open chromatin were more accessible for DNA methylase machinery and therefore they were more preferred targets of methylation. This hypothesis was even further confirmed by decreased expression of a transcriptional repressor TRIM28 and hypermethylation of its sites. The opposite effect was detected for HDAC6 binding sites: they were the only epigenetic features which showed overall DNA demethylation rather than hypermethylation. This also supports the formulated hypothesis as increased expression of an epigenetic repressor HDAC6 coincided with hypomethylation of its sites.

## Methods

### Cell culture

Caki-1 cell line was grown in Dulbecco’s modified Eagle medium supplemented with 10% fetal bovine serum, 1% penicillin/streptomycin, and 2 mM L-glutamine. Cells were transfected by Lipofectamine 3000 (Thermo Fisher Scientific) according to the manufacturer’s recommendation.

### CRISPR/Cas9-based gene editing

VHL gene editing with CRISPR/Cas9 was performed as described in our previous paper^7^. We introduced a homozygous frameshift mutation which led to a stop loss in the C-terminal alpha domain of VHL. Sanger sequencing confirmed that on the protein level, the original sequence VRSLYE(Stop) was substituted by VRSLYESGRPPKCAERPGAADTGAHCTSTDGRKLISVETYTVSSOLLMetVLMetSLDLDTGLVPSLVSKCLILRVK(Stop).

### DNA methylation analysis

Three individual biological replicates from Caki-1 cell line and VHL* clone were taken for bisulfite sequencing. DNA methylation was profiled by reduced representation bisulfite sequencing (RRBS). Two micrograms of genomic DNA from a sample were digested using 60 U MspI (Fermentas, USA) in 50 μl at 37 °С for 18–24 h, followed by QIAquick purification (Qiagen, Germany). The end of the digested DNA was repaired, and an adenine was added to the 3′ end of the DNA fragments according to the Illumina standard end repair and add_A protocol (Illumina, USA). Pre-annealed forked Illumina adaptors containing 5′- methylcytosine instead of cytosine were ligated to both ends of DNA fragments using standard Illumina adaptor ligation protocol (Illumina, USA). Ligated fragments were then separated by 2% agarose gel (Sigma-Aldrich, USA). Fragments between 170 bp and 350 bp, (includes adaptor length), were selected and cut from the gel. DNA from gel slices were purified using the Qiagen Gel Extraction Kit (Qiagen, Germany). The sodium bisulfite treatment and subsequent clean-up of size selected DNA was performed with the EZ DNA Methylation^TM^ Kit (ZymoResearch, USA) according to the manufacturer’s instructions. The bisulfite-treated DNA fragments were amplified using PCR and the following reaction: 5 μl of eluted DNA, 1 μl of NEB PE PCR two primers (1.0 and 2.0) and 45 μl Platinum PCR Supermix (Invitrogen, USA). The amplification conditions were as follows: 5 min at 95 °C, 30 s at 98 °C then 15 (10 s at 98 °C, 30 s at 65 °C, 30 s at 72 °C), followed by 5 min at 72 °C. The PCR reaction was purified by MinElute PCR Purification Kit (Qiagen), and final reduced representation bisulfite library was eluted in 15 μl EB buffer. The concentration of the final library was measured using the Agilent 2100 Bioanalyzer (Agilent Technologies, USA). The library was sequenced on Illumina 2500 platform according to standard Illumina cluster generation and sequencing protocols. 100-bp single-end reads were generated.

Reads were mapped to hg19 reference genome with Bismark software^15^.

Differential methylation analysis was performed by MethPipe/RADmeth software package^16,17^. It is one of the tools for bisulfite-seq analysis which account for the fact that they are dealing with fractions of counts (e.g., for a particular CpG position, x reads support DNA methylation of the total of N reads covering the position). The tool operates with counts rather than with methylation rates (x/N) which are essentially prone to biases due to different coverage of a given position in each sample. It models the counts with beta-binomial distribution thus accounting for both binomially distributed shot noise in read counts and biological variance in DNA methylation of a given position between samples: *k* ~ *Binomial*(*N*, *p*), where *p* ~ *Beta* (*α*, *β*), and *α* and *β* are fitted parameters. MethPipe/RADmeth was applied to bisulfite alignments as described in the manual. DNA methylation counts were merged between the forward and the reverse strand for each CpG thus excluding potentially mutated CpG sites. We first found differentially methylated individual CpG positions. Next, the positions were aggregated to differentially methylated regions (DMRs) with the default parameters.

ENSEMBL gene annotation (version 81) was used to find genes with TSS localized no further than 1 kilobase from differentially methylated regions.

### Publicly available DNA methylation data

DNA methylation data for four kidney cancer cell lines (A498, CAKI-1, 786-O, ACHN) profiled with Illumina 450 K array were taken from GSE49143 GEO dataset. Kidney cancer samples profiled with Illumina 450 K array were taken from KIRC TCGA cohort.

### RNA-seq analysis

For RNA-seq, we used the same sample collection and treatment procedure as described for bisulfite sequencing. Four fish from each of the four experimental groups were taken for transcriptome analysis. Gills were isolated and fixed with IntactRNA® reagent (Evrogen).

Total RNA was extracted from the samples with Trisol reagent according to the manufacturer’s instructions (Invitrogen). Quality was checked with the BioAnalyser and RNA 6000 Nano Kit (Agilent). PolyA RNA was purified with Dynabeads® mRNA Purification Kit (Ambion). An Illumina library was made from polyA RNA with NEBNext® mRNA Library Prep Reagent Set (NEB) according to the manual. Paired-end sequencing was performed on HiSeq. 1500 with 2 × 75 bp read length. Approximately 25 million reads were generated for each sample.

Reads were mapped to hg19 genome with tophat2 software (version 2.1.0)^18^. Gene models of non-overlapping exonic fragments were taken from ENSEMBL 54 database. For each exonic fragment, total coverage by mapped reads in each sample was calculated with bedtools multicov tool (version 2.17.0). Total gene coverage was calculated as a sum of coverages of all non-overlapping exonic fragments of a gene. Differential expression analysis was performed by applying default read count normalization (estimateSizeFactors) and performing per-gene negative binomial tests (nbinomTest), implemented in DESeq R package (version 1.22.0), with default parameters^19^. For each gene, the package provided both p-values and FDRs (p-values after Benjamini-Hochberg multiple testing corrections).

### Transcription factor motifs

PWM for HIF1a binding motif was downloaded from HOCOMOCO database^20,21^. Occurrences of HIF1a binding motif were searched in hg19 genome with SARUS software^22^.

### Epigenomics tracks

Epigenomic tracks for hg19 genome were downloaded from ENCODE for HepG2 and GM12878 cell line. In particular, we studied peaks histone marks (wgEncodeBroadHistone tables), ChromHMM genome segmentation track for HepG2 cell line, and clustered transcription factor binding sites, conserved across many cell lines (wgEncodeRegTfbsClusteredV3).

## Electronic supplementary material

Supplementary figures
