# Supplementary material for: VHL inactivation without hypoxia is sufficient to achieve genome hypermethylation
Source: Sci Rep. 2018 Jul 13;8:10667. doi: 10.1038/s41598-018-28795-y (PMC6045645; doi:10.1038/s41598-018-28795-y)
Supplement: Supplementary file 1 — Supplementary figures [file 41598_2018_28795_MOESM1_ESM.pdf]

VHL inactivation without hypoxia is sufficient to achieve genome hypermethylation

Artem V. Artemov[1*], Nadezhda Zhigalova[1], Svetlana Zhenilo[1], Alexander M. Mazur[1] and Egor B. Prokhortchouk[1*]

[1] Institute of Bioengineering, Research Center of Biotechnology RAS, Moscow, Russia

*** Correspondence to: artem.v.artemov@gmail.com, prokhortchouk@gmail.com**

Supporting Information

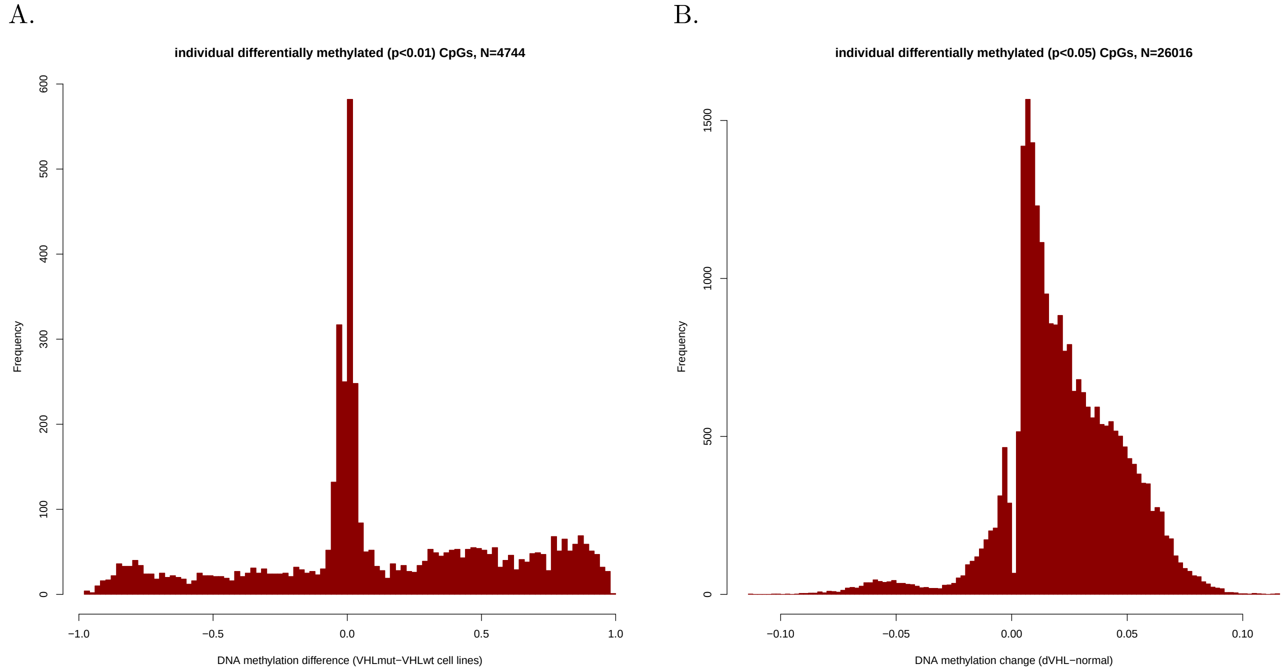

Figure S1. Similar to Figure 2. Distribution of changes in DNA methylation rates at individual CpGs following VHL inactivation. Only CpG positions which significantly changed their DNA methylation level were plotted. (A) Methylation difference between renal cancer cell lines with mutated VHL (A498, 786-O) and with wild-type VHL (ACHN, Caki-1). DNA methylation is on average higher in cell lines with mutated VHL ($p < 2 * 10^{-16}$ for all CpGs). (B) TCGA kidney tumours with mutated VHL show increase in DNA methylation as compared to kidney tumors with wild-type VHL ($p < 2 * 10^{-16}$ for all CpGs). Tumours with mutated SETD2 gene were excluded from this analysis.

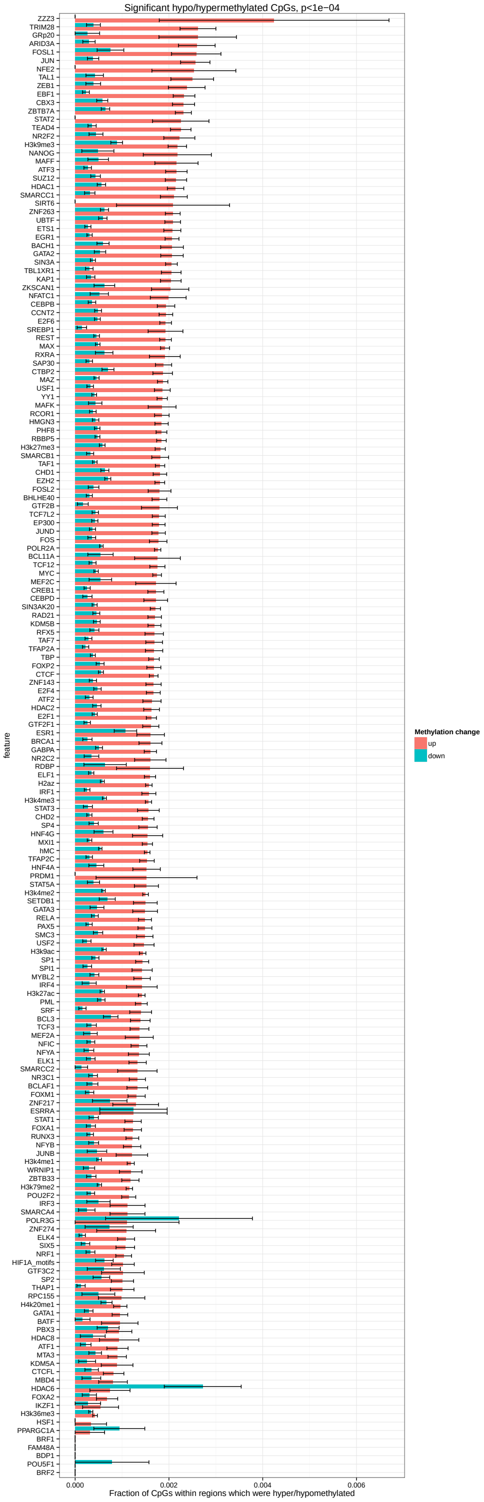

Figure S2. Full version of figure 4. Distribution of hypo- and hypermethylated CpGs within certain epigenomic features. Fraction of significantly hypo- (blue bars) and hypermethylated (red bars) CpGs among all CpGs within a given set of transcription factor binding sites. CpGs that were significantly hypermethylated after VHL inactivation, were enriched in AP-1 (JUN/FOS) and TRIM28 binding sites. Hypomethylated CpGs were enriched in HDAC6 binding sites.

A. B.

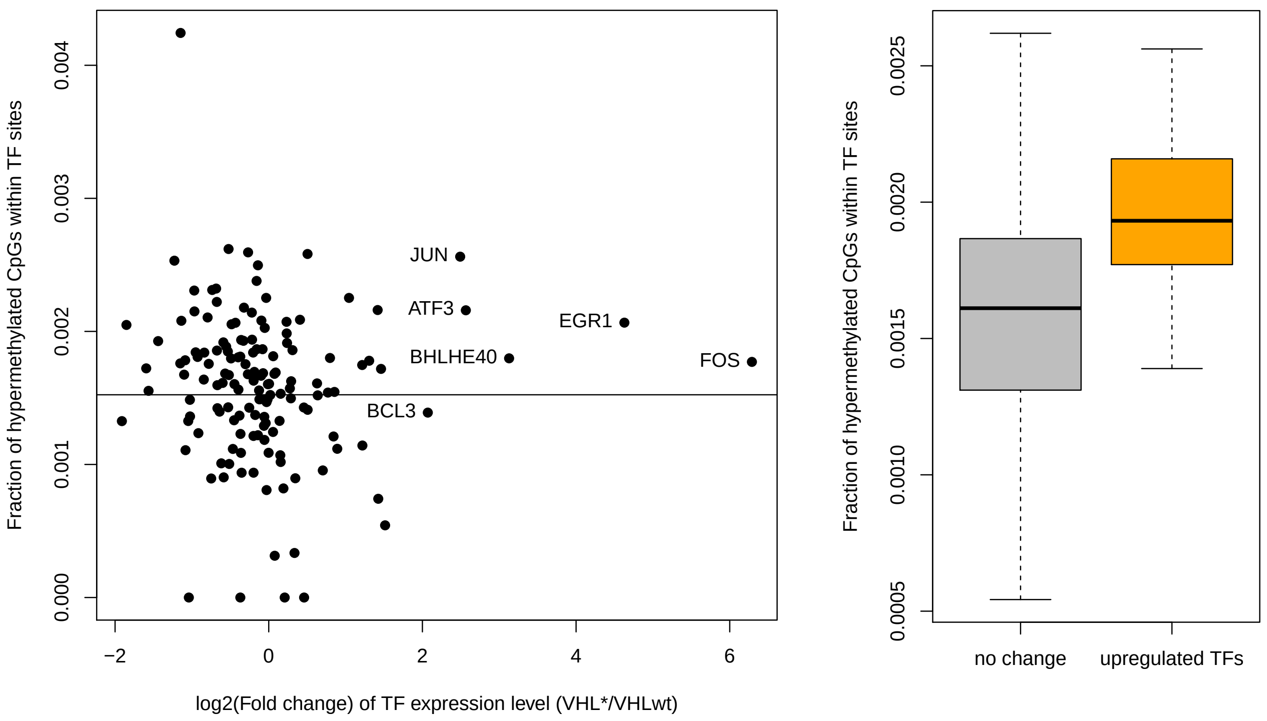

Figure S3. (A) Fraction of significantly hypermethylated CpGs (in Caki-1 VHL* vs Caki-1 cells) among all CpGs within sites of a certain DNA binding protein plotted against of gene expression change (between Caki-1 VHL* and Caki-1 cells) of a given DNA binding protein. Horizontal line indicates mean fraction of significantly hypermethylated CpGs among all DNA binding sites. (B) DNA binding genes that were more than twofold upregulated in VHL* show slightly higher rate of hypermethylation in their binding sites ($P = 0.6$).
